# A cohort study on the evolution of psychosocial problems in older patients with breast or colorectal cancer: comparison with younger cancer patients and older primary care patients without cancer

**DOI:** 10.1186/s12877-015-0071-7

**Published:** 2015-07-09

**Authors:** Laura Deckx, Doris L. van Abbema, Marjan van den Akker, Carine van den Broeke, Mieke van Driel, Paul Bulens, Vivianne C.G. Tjan-Heijnen, Cindy Kenis, Eric T. de Jonge, Bert Houben, Frank Buntinx

**Affiliations:** Department of General Practice, KU Leuven, Kapucijnenvoer 33, bus 7001, 3000 Leuven, Belgium; Department of Medical Oncology, GROW – School for Oncology and Developmental Biology, Maastricht University Medical Centre, PO Box 5800, 6202 AZ Maastricht, The Netherlands; Department of Family Medicine, CAPHRI - School for Public Health and Primary Care, Maastricht University Medical Centre, P.O. Box 616, 6200 MD Maastricht, The Netherlands; Discipline of General Practice, School of Medicine, The University of Queensland, Building 16/910, Royal Brisbane and Women’s Hospital, Brisbane, 4029 QLD Australia; Limburgs Oncologisch Centrum, Stadsomvaart 11, 3500 Hasselt, Belgium; Department of General Medical Oncology, University Hospitals Leuven, UZ Herestraat 49 - box 815, 3000 Leuven, Belgium; Department of Gynaecology, Ziekenhuis Oost-Limburg, Schiepse Bos 6, 3600 Genk, Belgium; Department of Abdominal and Oncological Surgery, Jessa Hospital – Campus Salvator, Salvatorstraat 20, 3500 Hasselt, Belgium

**Keywords:** Depression, Fatigue, Cognition, Oncology, Older cancer patients, Ageing

## Abstract

**Background:**

Although older cancer survivors commonly report psychosocial problems, the impact of both cancer and ageing on the occurrence of these problems remains largely unknown. The evolution of depression, cognitive functioning, and fatigue was evaluated in a group of older cancer patients in comparison with a group of younger cancer patients and older persons without cancer.

**Methods:**

Older (≥70 years) and younger cancer patients (50 – 69 years) with breast or colorectal cancer stage I - III, and older persons without cancer (≥70 years) were included. Data were collected at baseline and one year follow-up and were available for 536 persons. Depression was evaluated with the 15-item Geriatric Depression Scale. Cognitive functioning was measured with the cognitive functioning subscale of the European Organization for Research and Treatment of Cancer. Fatigue was measured with a Visual Analogue Scale. Risk factors for depression, cognitive functioning, and fatigue were analysed using multivariate logistic regression analyses. Risk factors included cancer- and ageing-related factors such as functional status, cancer treatment, and comorbidities.

**Results:**

The evolution of psychosocial problems was similar for the group of older (*N* = 125) and younger cancer patients (*N* = 196): an increase in depression (*p* < 0.01), slight worsening in cognitive functioning (*p* = 0.01), and no clear change in fatigue. Also, compared to the group of people without cancer (*N* = 215), the differences were small and after one year of follow-up only depression was more frequent in older cancer patients compared to older persons without cancer (18 % versus 9 %, *p* = 0.04). In multivariate analyses the main risk factors for psychosocial problems after one year follow-up were changes in functional status and presence of baseline depression, fatigue, or cognitive impairment.

**Conclusion:**

Over the course of one year after a diagnosis of cancer, cancer patients face increasing levels of depression and increasing difficulties in cognitive functioning. The main risk factor for psychosocial problems was presence of the problem at baseline. This calls for regular screening for psychosocial problems and exchange of information on psychosocial functioning between different health care providers and settings during the treatment and follow-up trajectory of cancer patients.

**Electronic supplementary material:**

The online version of this article (doi:10.1186/s12877-015-0071-7) contains supplementary material, which is available to authorized users.

## Background

The number of cancer patients and cancer survivors is increasing and will continue to increase in the coming decades [1, 2]. Furthermore, not only the number of older persons that are diagnosed with cancer is high and increasing. The number of cancer survivors is increasing as well, due to the additional improvement in cancer treatment. Whereas cancer used to be a fatal disease, it is now developing towards a chronic or even curable disease [3]. A diagnosis and subsequent treatment of cancer can have a substantial and possibly long-lasting psychosocial impact on patients’ lives. This problem was brought to our attention by two reports of the Institute of Medicine in the US: the 2006 publication “From Cancer Patient to Cancer Survivor: Lost in Transition” and the 2008 publication “Cancer Care for the Whole Patient: Addressing Psychosocial Health Needs” [3, 4]. Psychosocial care for cancer patients and survivors has received increased attention since. In Belgium for example, hospitals receive additional funding for psychosocial care for cancer patients since 2008.

Despite growing attention for the issues cancer survivors face, only a limited number of initiatives and research projects specifically focus on older cancer patients and survivors. Nevertheless, the group of older cancer patients is the largest and fastest growing group of cancer patients. Currently, the mean age at cancer diagnosis is 66 years and 46 % of all new cancer patients are aged 70 years or older [5]. Despite representing such an important group, our knowledge of psychosocial problems in older cancer patients remains inadequate [6].

Previous research that focused on psychosocial problems in older cancer patients was mostly cross-sectional, and did not simultaneously compare older cancer patients with healthy controls or younger cancer patients. Hence, previous studies were unable to disentangle the mutual impact of both cancer and ageing, whereas both are likely to have a substantial impact on the patients’ psychosocial functioning.

With respect to the impact of cancer, research has often shown that older cancer survivors fare worse, physically as well as mentally, compared to older persons of the same age without cancer [7, 8]. A recent study found that older cancer patients indeed reported poorer physical functioning compared to healthy controls, but did not find support for a difference in psychological and cognitive functioning [6].

With respect to the impact of ageing, findings are inconsistent. Several studies show that younger cancer patients report more psychological problems compared to older cancer patients [9, 10]. However, older and younger cancer patients were similar with respect to the most common cancer-related symptoms such as lack of energy and difficulty concentrating [10]. Furthermore, the lower levels of psychosocial problems in older cancer patients may be due to less aggressive cancer treatments, and older patients might be more reluctant to report problems because they fear additional testing and treatments and they view their health problems as a normal part of ageing [10]. The measurement tools that were used might also play an important role, as these are not always adjusted for use in older persons. For example, the identification of depression in older persons asks for a somewhat different approach compared to younger persons as older persons less commonly disclose affective symptoms such as sadness, and instead tend to present with trouble concentrating and fatigue [11, 12].

To address psychosocial problems in older cancer patients, the evolution of depression, cognitive functioning, and fatigue was evaluated in a group of older cancer patients. Furthermore, the frequency and severity of these problems in older cancer patients was compared with younger cancer patients and with older persons without cancer. Finally, the influence of ageing- and cancer-related problems on the occurrence of depression, cognitive impairment, and fatigue after one year of follow-up was evaluated.

## Methods

The data for this study were collected as part of a prospective observational cohort study; the KLIMOP-study. The design of this study has been published previously [13]. Older cancer patients (OCP; ≥70 years), younger cancer patients (YCP; 50 – 69 years), and older primary care patients without a history of cancer, except non-melanoma of the skin (ONC; ≥70 years) were included. To date, there is no clear age cut-off or definition of an older (cancer) patient, in this study older was defined as a person aged 70 years and above [14]. In the present study, the included cancer patients were patients with an incident diagnosis of breast or colorectal cancer stage I – III. Cancer patients were recruited through seven hospitals in Belgium and the Netherlands within three months after cancer diagnosis. A priori sample size calculations yielded a total needed sample size between 50 and 313 participants per group (depending on the prevalence of for example depression in the control group), we aimed to recruit 320 patients per group per country, enabling within-country analyses [13].

ONC were recruited through general practices and home nurses in the same region. General practitioners asked all consecutive eligible patients (70 years and above, no history of cancer) to participate until 20 patients per general practitioner agreed to participate.

Exclusion criteria for OCP, YCP, and ONC were the inability to speak Dutch, a formal diagnosis of dementia, and an estimated life expectancy less than six months. Data of all patients with at least one year follow-up who were included in the study between June 2010 and August 2012 were used. At time of this study recruitment of patients was still ongoing.

### Data collection

Data collection and management were identical in the three groups. Data were collected through personal interviews or self-administered questionnaires at baseline (T0) and at one-year follow-up (T1). In one hospital, baseline data collection was integrated in a routine geriatric assessment. Therefore, data collection was slightly different in this hospital and did not include the same measurement tool for cognitive functioning and fatigue. For all patients, medical information was retrieved from the medical records of the hospital (for cancer patients) or the general practitioner (for older persons without cancer).

### Dependent variables

#### Depression

Depression was evaluated with the 15-item Geriatric Depression Scale (GDS-15), which was especially designed to screen for depression in an older population [15]. The total sum score ranges from 0 to 15. The mean score was used to indicate the severity of depression. A score of ≥5 was used as cut-off for the frequency of depression, for which sensitivity and specificity against a standard clinical interview have been shown to be 91 % and 72 % respectively. Furthermore, the GDS-15 has a high level of internal consistency (Cronbach’s alpha = 0.80) [16]. In our study, Cronbach’s alpha was 0.72 for the total population, 0.70 in OCP, 0.77 in YCP, and 0.68 in ONC.

#### Subjective cognitive functioning

Subjective cognitive functioning was measured with the cognitive functioning subscale of the quality of life questionnaire of the European Organization for Research and Treatment of Cancer (EORTC QLQ-C30) [17]. This subscale consists of two items (“During the past week have you had difficulty in concentrating on things, like reading a newspaper or watching television?” and “During the past week have you had difficulty remembering things?”). Based on the EORTC QLQ-C30 manual, cognitive functioning was recoded in a score ranging from 0 to 100, with higher scores representing better functioning [18]. The mean score was used to indicate the severity of cognitive impairment. The frequency of cognitive impairment was operationalized by using the lowest functioning quartile as cut-off, this corresponded to a score <67 in all three groups.

The internal consistency for the cognitive functioning subscale has been shown to be fair, with Cronbach’s alpha ranging between 0.52 and 0.73 [17]. In our study, Cronbach’s alpha was 0.47 for the total population, 0.57 in OCP, 0.65 in YCP, and 0.34 in ONC.

#### Fatigue

A Visual Analogue Scale (VAS) was used for measuring fatigue. Patients were asked: ‘On a scale of 0 to 10, how would you rate your fatigue over the past 24 h?’. The mean score was used to indicate the severity of fatigue. A cut-off ≥4 was used to define the frequency of fatigue.

### Covariables

#### Cancer-related variables

As cancer-related variables, cancer type (breast and colorectal cancer), cancer stage (I, II, III), and the cancer treatment modalities received in the first year after cancer diagnosis (surgery, chemotherapy, radiotherapy, hormonal therapy, and targeted therapy) were considered. As several combinations are possible, cancer treatment was operationalized according to physical impact: no treatment; surgery only; surgery and radiotherapy or hormonal therapy or both; surgery and chemotherapy with or without any combination of radiotherapy, hormonal therapy, and targeted therapy; other combinations. The group of ‘other’ cancer treatments consisted of people who received no surgery but instead any of the following combinations: chemotherapy; chemo- and radiotherapy; chemo- and targeted therapy; hormonal therapy.

Functional status is also a cancer-related factor, as cancer treatment often results in decline of functional status. However, for pragmatic reasons this is discussed under the ageing-related variables.

#### Ageing-related variables

As ageing-related factors, baseline age, comorbidity, number of drugs taken as chronic medication, and change in functional status between T0 and T1 were considered.

Comorbidity was measured with the Charlson Comorbidity Index (CCI), which provides a weighted score of 19 comorbid conditions [19]. The total score is derived by summing the assigned weights of all comorbid conditions. It ranges from 0 to 37 maximum; higher scores indicate multiple and/or more severe conditions. The mean CCI score was presented. In addition to the CCI, the mean number of drugs taken as chronic medication was also included.

Functional status was measured by Activities of Daily Living (ADL) using the Katz Index [20] and by Instrumental Activities of Daily Living (IADL) using the Lawton IADL-scale [21]. The Katz Index consists of six items and the total sum score ranges from 0 (dependent) to 6 (independent). The Lawton IADL-scale consists of eight items in women and five items in men. The total sum score ranges from 0 (dependent) to 8 (independent) in women, and from 0 (dependent) to 5 (independent) in men. Functional impairment was defined as dependency on at least one domain of ADL (score <6) or IADL (a score <8 in women or <5 in men). Change in functional status was operationalized as follows: not impaired (independent at T0 and T1), was impaired (dependent at T0; independent at T1), became impaired (independent at T0; dependent at T1), persistently impaired (dependent at T0 and T1).

#### Socio-demographic variables

Socio-demographic variables include sex (men versus women), living situation (living alone, with partner, with family or friends, institutionalized), and educational level (age at which they left school; <15, 15 – 18, >18 years).

### Ethics

The study protocol was approved by the Ethical Review Board of KU Leuven and UZ Leuven (S52097 – ML6279) (Belgium) and the Maastricht University Medical Centre (NL31414.068.10) (the Netherlands). All patients signed informed consent.

### Analysis

**Fig. 1:**
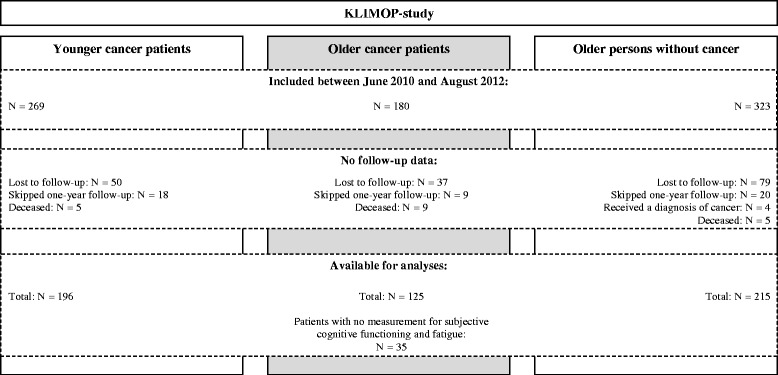
Flow-chart: patient population of the KLIMOP-study

Patients were excluded from analyses if they were lost to follow or deceased at T1, skipped data collection at T1, or received a diagnosis of cancer during follow-up (only applicable for ONC), for more detail see Fig. 1.

Socio-demographic and clinical characteristics of the study population are presented as the mean and standard deviation (SD) for continuous variables and as numbers and proportions for categorical variables. Comparisons between different groups were performed using the Wilcoxon-Mann-Whitney test for continuous data and the chi square test for categorical data. Comparisons within groups were performed using Wilcoxon signed ranks test for continuous data and the McNemar test for categorical data. A *p*-value < 0.05 was considered to be statistically significant throughout all analyses.

The relationship between independent and dependent variables at T1 was tested with multivariate logistic regression analyses. All analyses were adjusted for the baseline value of the dependent variable and for the same cancer- and ageing-related variables. Because the impact of both cancer and ageing on the dependent variables remains unknown, the possibility of interaction between age and cancer in the group of OCP and ONC and between age and cancer treatment in the group of OCP and YCP was evaluated. This was evaluated visually as well as statistically. Based on these analyses no indication for interaction between age and cancer or cancer treatment was found.

Due to colinearity between cancer treatment, type of cancer, and cancer stage, only the different combinations of cancer treatment were included in the model. The fit of the model was tested with the Hosmer and Lemeshow’s goodness-of-fit test.

As described above, the baseline measurement for cognitive functioning and fatigue was not collected for a subgroup of OCP for whom data collection was integrated in a routine geriatric assessment. Also, for some patients data on depression and functional status were missing. Therefore, a sensitivity analysis was performed to assess the influence of these missing values, making a worst- and best-case scenario by imputing missing values as either a normal or an abnormal score. Furthermore, the influence of sex and type of cancer was assessed by stratifying the analyses for these variables.

Statistical analyses were performed using the STATA statistical software package version 11 (StataCorp LP, College Station, TX).

## Results

### Description and follow-up of the population

Seven hundred and twenty-two patients were included in the KLIMOP-study between June 2010 and August 2012. During the one-year follow-up period, 9 OCP, 5 YCP, and 5 ONC died (*p* = 0.06 for OCP versus YCP; *p* = 0.03 for OCP versus ONC). Twenty-two percent were lost to follow-up and 6 % of the patients skipped data collection at T1 (see Fig. 1). The proportion of OCP and YCP that were lost to follow-up or skipped data collection at T1 was comparable (*p* = 0.79). The same was true for OCP versus ONC (*p* = 0.21).

For the outcome variables of interest, depression, cognitive functioning, and fatigue, baseline values for persons available for analyses were similar compared to those lost to follow-up (see Additional file 1: Table S1). Only the severity of depression was slightly higher for ONC who were lost to follow-up compared to those included in the analyses (*p* < 0.01), however the absolute difference was small (difference of 0.69) and the frequency of depression was comparable in both groups (*p* = 0.24).

For socio-demographic and clinical characteristics, OCP available for analyses were similar to those lost to follow-up. Only for cancer stage, the proportion of excluded OCP with stage II cancer was lower, and the proportion with stage III cancer was higher, compared to included OCP (*p* < 0.01). YCP and ONC available for analyses at T1 were comparable to those lost to follow-up (see Additional file 1: Table S1).

### Baseline characteristics

All patients were Caucasian, with the exception of two YCP. OCP and ONC were comparable with respect to age, living conditions, educational level, and functional status (see Table 1). The proportion of women was higher in OCP compared to ONC, due to the recruitment of breast cancer patients (*p* < 0.001). The mean CCI score, and mean number of drugs taken as chronic medication was higher for ONC compared to OCP (*p* < 0.01).

**Table 1:** Baseline population characteristics (N = 536)

	Younger cancer patients			Older cancer patients			Older persons without cancer	
	*N* = 196			*N* = 125			*N* = 215	
	N		*P* value^a^	N		*P* value^b^	N	
**Socio-demographic variables**								
*Gender*								
Male	28	14 %	0.33	23	18 %	0.00	77	36 %
Female	168	86 %		102	82 %		138	64 %
*Living situation*								
Alone	28	14 %	0.00	39	31 %	0.14	70	33 %
With partner	160	82 %		72	58 %		134	62 %
With friends/family	8	4 %		6	5 %		7	3 %
Institutionalized	0			8	6 %		4	2 %
*Age at leaving school*								
<15 years	18	9 %	0.00	33	26 %	0.72	67	31 %
15 – 18 years	95	48 %		60	48 %		98	46 %
>18 years	76	39 %		28	22 %		48	22 %
Missing	7	4 %		4	3 %		2	1 %
**Ageing-related variables**								
*Age* (years): mean ± SD	59.53	±5.30	0.00	77.18	±5.17	0.13	78.07	±5.41
*Number of drugs*: mean ± SD	2.29	±2.41	0.00	4.22	±3.45	0.01	5.07	±3.33
*CCI*: mean ± SD	0.30	±0.80	0.01	0.50	±0.70	0.00	1.36	±1.48
Missing	3			5			47	
*Functional status*								
Impaired	87	44 %	0.00	90	72 %	0.10	136	63 %
Missing	1	1 %		0	/		0	/
**Cancer-related variables**								
*Cancer site*								
Breast	150	77 %	0.66	93	74 %			
Colorectal	46	23 %		32	26 %			
*Cancer treatment*			0.00					
No treatment	0	0 %		1	1 %			
Surgery only	19	10 %		20	16 %			
Surgery and RT/HT or both	70	36 %		67	54 %			
Surgery and CT with or without any combination of RT/HT/TT	100	51 %		32	26 %			
Other ^c^	7	4 %		4	3 %			
Unknown	0	/		1	1 %			
*Cancer Stage*			0.00					
I	69	35 %		21	17 %			
II	75	38 %		71	57 %			
III	44	22 %		14	11 %			
Unknown	8	4 %		19	15 %			

*SD*, standard deviation; *CCI*, Charlson Comorbidity Index; *RT*, radiotherapy; *HT*, hormonal therapy; *CT*, chemotherapy; *TT*, targeted therapy

^a^Differences between older cancer patients and younger cancer patients

^b^Differences between older cancer patients and older persons without cancer

^c^Other cancer treatments consisted of people who received no surgery but instead any of the following combinations: chemotherapy only (*N* = 2), chemo- and radiotherapy (*N* = 6), chemo- and targeted therapy (*N* = 1), hormonal therapy only (*N* = 2)

For OCP versus YCP, the proportion of men and women was similar (*p* = 0.33), as well as the proportion of breast and colorectal cancer patients (*p* = 0.66). OCP differed from YCP with respect to age, living situation, educational level, functional status, CCI, number of drugs taken as chronic medication, and cancer treatments received (see Table 1). OCP and YCP also differed for cancer stage at diagnosis. However, when stratified according to cancer type, this was only true for breast cancer patients (stage I: 21 % and 45 %; stage II: 74 % and 44 %; stage III: 5 % and 12 % respectively).

### Influence of ageing and cancer

**Fig. 2:**
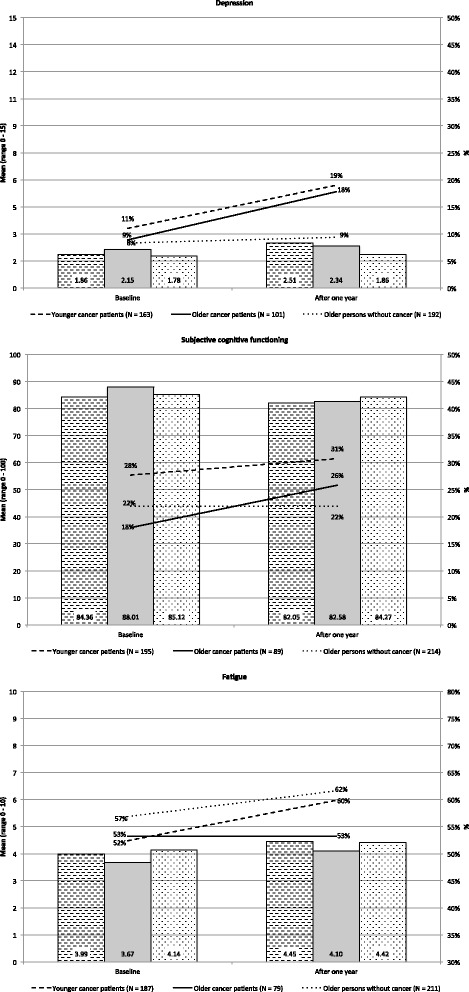
Frequency and severity of psychosocial problems at baseline and one-year follow-up. *Note:* Depression was measured with the 15-item Geriatric Depression Scale, range 0 – 15, higher scores indicate more depression, cut-off ≥5 for frequency of depression. Cognitive functioning was measured with the cognitive functioning subscale of the EORTC QLQ-C30, range 0 – 100, lower scores indicate worse functioning, cut-off <67 for frequency of cognitive impairment. Fatigue was measured with a Visual Analogue Scale, range 0 – 10, higher scores indicate more fatigue, cut-off ≥4 for frequency of fatigue

Figure 2 shows the severity and frequency of the selected problems in the three groups at T0 and T1. Details of the differences between and within groups are provided in Table 2.

**Table 2:** Frequency and severity of psychosocial problems at baseline and one-year follow-up

	Younger cancer patients		*P* value between group difference^a^	Older cancer patients		*P* value between group difference^b^	Older persons without cancer	
**DEPRESSION:**	*N* = 163			*N* = 101			*N* = 192	
**Severity:** ** mean (±SD)**								
Baseline	1.86	(±2.28)	0.10	2.15	(±2.30)	0.10	1.78	(±2.03)
After one year	2.51	(±2.85)	0.77	2.34	(±2.29)	0.10	1.86	(±2.09)
*P* value within group difference	0.02			0.35			0.58	
**Frequency:** ** N (%)**								
Baseline	18	(11 %)	0.58	9	(9 %)	0.87	16	(8 %)
After one year	31	(19 %)	0.81	18	(18 %)	0.04	18	(9 %)
*P* value within group difference	0.02			0.04			0.80	
**COGNITIVE FUNCTIONING:**	*N* = 195			*N* = 89			*N* = 214	
**Severity:** ** mean (±SD)**								
Baseline	84.36	(±22.36)	0.40	88.01	(±17.59)	0.04	85.12	(±16.41)
After one year	82.05	(±21.27)	0.90	82.58	(±19.28)	0.65	84.27	(±17.07)
*P* value within group difference	0.10			0.01			0.48	
**Frequency: N (%)**								
Baseline	54	(28 %)	0.08	16	(18 %)	0.44	47	(22 %)
After one year	60	(31 %)	0.40	23	(26 %)	0.47	47	(22 %)
*P* value within group difference	0.51			0.14			1.00	
**FATIGUE:**	*N* = 187			*N* = 79			*N* = 211	
**Severity: mean (±SD)**								
Baseline	3.99	(±2.82)	0.41	3.67	(±2.77)	0.17	4.14	(±2.30)
After one year	4.45	(±2.66)	0.30	4.10	(±2.49)	0.30	4.42	(±2.31)
*P* value within group difference	0.08			0.24			0.12	
**Frequency: N (%)**								
Baseline	98	(52 %)	0.91	42	(53 %)	0.57	120	(57 %)
After one year	112	(60 %)	0.31	42	(53 %)	0.19	130	(62 %)
*P* value within group difference	0.12			1.00			0.26	

Depression, cognitive functioning, and fatigue are presented as the mean score – indicated as the severity – and the proportion – indicated as frequency. Depression was measured with the 15-item Geriatric Depression Scale, range 0 – 15, higher scores indicate more depression, cut-off ≥5 for frequency of depression. Cognitive functioning was measured with the cognitive functioning subscale of the EORTC QLQ-C30, range 0 – 100, lower scores indicate worse functioning, cut-off <67 for frequency of cognitive impairment. Fatigue was measured with a Visual Analogue Scale, range 0 – 10, higher scores indicate more fatigue, cut-off ≥4 for frequency of fatigue

^a^Differences between older cancer patients and younger cancer patients

^b^Differences between older cancer patients and older persons without cancer

#### Depression

The frequency of depression increased significantly from 9 % to 18 % in OCP (*p* = 0.04) and from 11 % to 19 % in YCP (*p* = 0.02), while it remained stable in ONC (*p* = 0.80). At T1, the frequency of depression was significantly higher in OCP compared to ONC (*p* = 0.04), but the severity was similar for OCP and ONC (*p* = 0.10). For OCP and YCP, the severity and frequency of depression was similar at T0 and T1 (see Fig. 2 and Table 2).

Multivariate analyses showed that cancer treatment, especially the combination of surgery with chemotherapy, significantly increased the risk of depression (OR: 3.26; 95 % CI: 1.20 - 8.87), while ageing-related factors such as chronological age, number of drugs taken as chronic medication, and comorbidity were not associated with depression (see Table 3). Changes in functional status were associated with depression. People who became impaired (OR: 3.99; 95 % CI: 1.14 - 13.90) or were persistently impaired (OR: 7.78; 95 % CI: 2.60 - 23.29) in functional status were almost four and eight times more likely to be depressed at T1 compared to people who were not impaired. Other factors that predicted the occurrence of depression were depression at T0 (OR: 10.39; 95 % CI: 4.65 - 23.22) and living alone (OR: 2.70; 95 % CI: 1.31 - 5.56).

**Table 3:** Factors associated with psychosocial problems at one-year follow-up

	Depression at T1		Cognitive functioning at T1		Fatigue at T1	
	N = 421		N = 429		N = 413	
	OR	(95 % CI)	OR	(95 % CI)	OR	(95 % CI)
*Baseline value of the problem*	10.39	(4.65 - 23.22)	4.21	(2.56 - 6.93)	3.53	(2.20 - 5.64)
**Ageing-related variables**						
*Age:* mean	0.97	(0.93 - 1.01)	1.00	(0.97 - 1.04)	1.00	(0.97 - 1.03)
*Number of drugs:* mean	1.08	(0.97 - 1.20)	1.03	(0.94 - 1.12)	1.16	(1.06 - 1.27)
*Comorbidity:* mean CCI score	1.10	(0.80 - 1.50)	0.96	(0.77 - 1.20)	1.18	(0.92 - 1.51)
*Change in functional status*						
Not impaired	1	Reference	1	Reference	1	Reference
Was impaired	2.56	(0.69 - 9.54)	1.42	(0.62 - 3.25)	1.41	(0.67 - 2.94)
Became impaired	3.99	(1.14 - 13.90)	1.91	(0.86 - 4.24)	2.14	(1.04 - 4.43)
Persistently impaired	7.78	(2.60 - 23.29)	2.34	(1.21 - 4.54)	1.17	(0.65 - 2.11)
**Socio-demographic variables**						
*Gender* (women)	0.56	(0.26 - 1.24)	0.91	(0.49 - 1.66)	2.08	(1.16 - 3.73)
*Living conditions*						
With partner	1	Reference	1	Reference	1	Reference
With friends/family	2.54	(0.63 - 10.26)	1.08	(0.34 - 3.45)	0.87	(0.28 - 2.66)
Institutionalised	2.02	(0.30 - 13.69)	0.28	(0.03 - 2.86)	0.63	(0.09 - 4.20)
Alone	2.70	(1.31 - 5.56)	1.10	(0.61 - 1.97)	0.97	(0.55 - 1.71)
*Age at leaving school*						
<15 years	1	Reference	1	Reference	1	Reference
15 – 18 years	0.91	(0.41 - 2.02)	1.18	(0.62 - 2.26)	1.95	(1.06 - 3.61)
>18 years	0.80	(0.32 - 2.02)	1.18	(0.58 - 2.40)	1.44	(0.74 - 2.80)
**Cancer-related variables**						
*Cancer treatment*						
No cancer	1	Reference	1	Reference	1	Reference
Surgery only	1.35	(0.31 - 5.76)	0.87	(0.29 - 2.61)	0.44	(0.17 - 1.15)
Surgery and RT/HT therapy or both	2.48	(0.97 - 6.36)	1.59	(0.76 - 3.32)	0.77	(0.39 - 1.53)
Surgery and CT with or without any combination RT, HT, TT	3.26	(1.20 - 8.87)	2.17	(0.99 - 4.78)	1.73	(0.80 - 3.72)
Other^a^	/		1.28	(0.21 - 7.74)	0.82	(0.15 - 4.36)
Percentage correctly classified	86 %		76 %		71 %	
Goodness-of-fit test	0.90		0.18		0.30	

*OR*, Odds Ratio; *95 % CI*, 95 % Confidence Interval; *CCI*, Charlson Comorbidity Index; *RT*, radiotherapy; *HT*, hormonal therapy; *CT*, chemotherapy; *TT*, targeted therapy

^a^Other cancer treatments consisted of people who received no surgery but instead any of the following combinations: chemotherapy only (*N* = 2), chemo- and radiotherapy (*N* = 6), chemo- and targeted therapy (*N* = 1), hormonal therapy only (*N* = 2)

#### Cognitive functioning

The frequency and severity of cognitive impairment increased slightly in both cancer groups, although the difference was only significant for the severity of cognitive functioning in OCP (*p* < 0.01) (see Table 2). In ONC cognitive impairment remained stable. At both T0 and T1, OCP and YCP were comparable with respect to frequency and severity of cognitive impairment. OCP reported better cognitive functioning compared to ONC at T0 (*p* = 0.04).

Multivariate analyses showed that only impaired cognitive functioning at T0 (OR: 4.21; 95 % CI: 2.56 - 6.93) and persistent impairment in functional status (OR: 2.34; 95 % CI: 1.21 - 4.54) predicted cognitive impairment at T1 (see Table 3).

#### Fatigue

The frequency of fatigue remained stable in OCP at 53 %, in YCP it increased from 52 % to 60 %, and in ONC the frequency of fatigue increased from 57 % to 62 %. However, this increase was not significant, neither in YCP (*p* = 0.12), nor in ONC (*p* = 0.26). Similarly, the severity of fatigue increased slightly, but not significantly. At both time points, the frequency and severity of fatigue was similar for OCP and YCP, and for OCP and ONC.

In multivariate analyses the association between cancer treatment and occurrence of fatigue was not significant (see Table 3). For functional status, results showed that especially people who became impaired were at risk of fatigue (OR: 2.14; 95 % CI: 1.04 - 4.43). One other ageing-related factor that predicted fatigue was the increasing number of drugs taken as chronic medication (OR: 1.16; 95 % CI: 1.06 - 1.27). In line with the previous analyses, also presence of fatigue at T0 significantly predicted fatigue at T1 (OR: 3.53; 95 % CI: 2.20 - 5.64). Other factors that were associated with increased risk of fatigue were being female (OR: 2.08; 95 % CI: 1.16 - 3.73) and leaving school at age 15 – 18 years compared to leaving school at age <15 years (OR: 1.95; 95 % CI: 1.06 - 3.61).

### Sensitivity analyses

Imputing missing values as either best- or worst-case scenario did not change the conclusion of our results. For some variables such as the number of drugs taken as chronic medication and the association with cancer therapy, the worst- and best-case scenarios tipped the balance to significance. However, the lower level of the 95 % confidence interval was in this case never higher than 1.01 (see Additional file 2: Table S2).

When the evolution of severity and frequency of depression was stratified according to sex, OCP, especially women experienced a significant increase in depression from 7 % at T0 to 19 % at T1 (*p* < 0.001) (see Additional file 3: Table S3). For male OCP were not able to show a difference, however numbers were small (*N* = 18). Also for YCP both men and women experienced an increase in depression. The results for men need to be interpreted with caution as the number of men was small (*N* = 26) and at baseline only one person reported depression. For ONC, depression did not change neither in men nor women. For cognitive functioning and fatigue results remained similar when stratified according to sex. Cognitive impairment increased slightly in the two cancer groups, which was only significant in female OCP (*p* < 0.001). Again, the group of male cancer patients was too small to formulate reliable conclusions.

The evolution of depression, cognitive impairment, and fatigue followed the same trend in breast and colorectal cancer patients and the frequency and severity was similar between breast and colorectal cancer patients at all time points (see Additional file 4: Table S4).

## Discussion

In this paper, the occurrence of psychosocial problems in OCP at time of cancer diagnosis and one year later was studied. The frequency of depression increased and cognitive functioning slightly worsened in the group of OCP as well as YCP. However, OCP were not at higher nor lower risk of depression, impaired cognitive functioning, and fatigue compared to YCP. Compared to ONC, the difference in cognitive functioning and fatigue was small, and after one year of follow-up, only depression was more frequent in OCP compared to ONC. In multivariate analyses, the main risk factors for psychosocial problems were changes in functional status and presence of baseline depression, fatigue or cognitive impairment.

### An important timeframe

The occurrence of psychosocial problems was studied at time of cancer diagnosis and one year after a diagnosis of cancer. This is a timeframe that often corresponds with the transition from secondary to primary care, i.e. the time when general practitioners (GPs) become the first contact person again, especially for psychosocial issues. Previous studies emphasised that the transition from secondary to primary care is associated with increased psychological distress [22, 23]. Our study also showed more depression and some worsening of cognitive functioning one year after cancer diagnosis. While psychosocial care might be well organized around cancer diagnosis and treatment within the hospital setting, there is no consensus yet on the organization of cancer aftercare [24]. Whether or not GPs will claim a formal role in cancer aftercare, their role will become more prominent due to the increasing numbers of OCP and the shift from inpatient to ambulatory care [25]. Hence, GPs but also other primary and secondary health care providers should be aware of the psychosocial problems cancer patients may encounter, also when primary cancer treatment has ended. Furthermore, presence of the problem at baseline was the main risk factor for psychosocial problems one year after cancer diagnosis. This highlights the importance of regular screening for psychosocial problems and exchange of information on psychosocial functioning between secondary and primary care professionals.

### The impact of ageing: older cancer patients versus younger cancer patients

Increasing age is associated with the accumulation of personal and health-related losses and diminishing financial and social resources, which may contribute to increased vulnerability [26]. Therefore, it is not unlikely that OCP may face more psychosocial problems compared to YCP, e.g. more depression and more cognitive problems.

Two cross-sectional studies showed that within the group of OCP, increasing age was positively associated with depression [26, 27]. Although there was no association with chronological age in this study, multivariate analyses showed that several ageing-related problems were associated with psychosocial problems. For example, becoming impaired or being persistently impaired on functional status predicted all three selected psychosocial problems, including depression. Also the mean number of drugs taken as chronic medication predicted the occurrence of fatigue and people who were living alone were at increased risk for depression. As such, adequate (social) support seems an essential element in safeguarding psychosocial wellbeing of older people. Adequate support may countervail the negative effects of functional impairment, comorbidity, and living alone.

When comparing OCP and YCP, this study showed that the occurrence of psychosocial problems was similar in both groups at time of cancer diagnosis and one year later. These findings are consistent with those from a recent cross-sectional study among older (≥60 years) and younger (<60 years) patients undergoing cancer treatment [10]. Another study showed that the level of depression was comparable in older (≥65 years) and younger (45 – 65 years) cancer patients 3 months, 15 months, and 8 years after cancer diagnosis [28].

However, in contrast to our findings, several other studies reported better psychosocial functioning in OCP compared to YCP (see for example [9, 10, 29]). The definition of ’older’ and ‘younger’ patients might explain these inconsistent results as this varies widely across different studies [26]. Cohen et al. emphasize that studies showing a better functioning in OCP often used a cut-off for old age at relatively young age; 50 or 60 years [26]. Until now, there is no clear consensus on the definition of an OCP. However, the International Society of Geriatric Oncology uses 70 years and above as age cut-off for an OCP [14].

### The impact of cancer: older cancer patients versus older persons without cancer

OCP often fare worse, physically as well as mentally, compared to people of the same age without cancer [7, 8]. In this study, the prevalence of depression at one-year follow-up in OCP was twice the percentage of ONC, and also cancer patients who received chemotherapy were at increased risk for depression compared to people without cancer. However, for cognitive functioning and fatigue, OCP and ONC were not different after one year of follow-up. In contrast with our results, a recent study among long-term cancer survivors (15 years since diagnosis) showed no difference between OCP and healthy controls with respect to depression [6]. A possible explanation for these different findings is that the first year after diagnosis is strongly affected by the consequences of cancer and its treatment, while in the long term psychological functioning of cancer survivors may be more affected by ageing [28]. In contrast, we could not show a significant association between cancer treatment and fatigue or subjective cognitive functioning. Fatigue may be secondary to the physical and psychological stress associated with cancer and its treatment [30]. Furthermore, comorbidity may play an important role in the occurrence of fatigue; the drugs as well as the underlying comorbidity may contribute to the occurrence of fatigue [30, 31]. This may explain why the prevalence fatigue was not higher in cancer patients compared to people without cancer. In our study, comorbidity as measured by the CCI was not significantly associated with fatigue, but the number of drugs taken as chronic medication significantly predicted the occurrence of fatigue. However, it was beyond the scope of this study to investigate the role of specific cancer treatment regimens on cognitive functioning or fatigue.

### Strengths and limitations

The key strengths of this study are its longitudinal design, the two control groups that allow us to disentangle the effects of ageing and cancer, and the use of measurement instruments that are appropriate in older persons. Nevertheless, this study has some limitations. For example, the GDS-15, a well-validated screening instrument for depression in an older (cancer) population [11], may not be as suitable for YCP. However, we do not believe this biased our results. The GDS was designed to reduce the focus on somatic symptoms of depression [15]. This is an important feature for the identification of depression in cancer patients. Identification of depression in cancer patients in especially challenging because the symptoms of depression are often similar to those of somatic diseases or their treatment [32]. Furthermore, Cronbach’s alpha was also high for YCP (0.77). In contrast we found relatively low Cronbach’s alpha for subjective cognitive functioning, in particular in older persons without cancer. Hence results with respect to subjective cognitive functioning should be interpreted with caution. Nonetheless, Cronbach’s alpha was relatively low in the two groups of cancer patients as well and also previous studies in cancer patients have reported a wide range of Cronbach’s alphas for this subscale [33]. Another limitation is loss to follow-up and incomplete data. Twenty-two percent of patients were lost to follow-up. This is comparable with other large health and ageing studies in Europe (e.g. 22 % loss to follow-up in the English Longitudinal Study of Ageing) [34]. Furthermore, patients lost to follow-up were comparable to those available for analyses and our results were robust for imputing yes or no for missing values in a sensitivity analysis. Loss to follow-up may jeopardise the power of the study, for example the confidence intervals for depression were wide. This lack of precision should be taken into account when generalizing the results. With respect to the influence of sex, the generalizability of our results may be limited for men, because the majority of cancer patients in this study were women. It is known, for example, that women are more likely to report feelings of depression compared to men [35]. When stratified according to sex, the increase in depression was indeed only apparent in female OCP. However, we were not able to formulate any conclusions on male patients as a separate group, as the number of men was limited. Additionally, also the selection of breast and colorectal cancer patients with a relatively good prognosis needs to be considered when generalizing these results.

## Conclusion

This study showed that OCP as well as YCP face increasing levels of depression and increasing difficulties in cognitive functioning over the course of one year after a diagnosis of cancer, a timeframe that corresponds with the transition from secondary to primary care. The main risk factor for psychosocial problems was presence of the problem at baseline. Hence, health care providers should be aware of the psychosocial problems cancer patients may encounter, also when primary cancer treatment has ended. Furthermore, this highlights the importance of regular screening for psychosocial problems and exchange of information on psychosocial functioning between different health care providers and settings during the treatment and follow-up trajectory of cancer patients. More longitudinal studies with similar control groups are needed in order to confirm our results.

## Additional files

Additional file 1: Table S1. Baseline characteristics of persons available for analyses versus those lost to follow-up or those who skipped data collection at T1 (N = 749). Additional file 2: Table S2. Sensitivity analyses: best-case and worst-case scenario. Additional file 3: Table S3. Frequency and severity of psychosocial problems at baseline and one-year follow-up for men and women separately. Additional file 4: Table S4. Frequency and severity of psychosocial problems at baseline and one-year follow-up for breast and colorectal cancer patients separately.

## Abbreviations

ADL: Activities of Daily Living
CCI: Charlson Comorbidity Index
GDS-15: 15-item Geriatric Depression Scale
IADL: Instrumental Activities of Daily Living
OCP: Older cancer patients
ONC: Older primary care patients without a history of cancer
SD: Standard deviation
T0: Baseline (at time of cancer diagnosis)
T1: After one year of follow-up
VAS: Visual Analogue Scale
YCP: Younger cancer patients
