# Supplementary material for: A cohort study on the evolution of psychosocial problems in older patients with breast or colorectal cancer: comparison with younger cancer patients and older primary care patients without cancer
Source: BMC Geriatr. 2015 Jul 9;15:79. doi: 10.1186/s12877-015-0071-7 (PMC4496825; doi:10.1186/s12877-015-0071-7)
Supplement: Additional file 1: Table S1. — Baseline characteristics of persons available for analyses versus those lost to follow-up or those who skipped data collection at T1 (N = 749). [file 12877_2015_71_MOESM1_ESM.docx]

**Additional file 1: Table S1.** Baseline characteristics of persons available for analyses versus those lost to follow-up or those who skipped data collection at T1 (N = 749)

|  | **Younger cancer patients** | | | | |  | **Older cancer patients** | | | | |  | **Older persons without cancer** | | | | |
| --- | --- | --- | --- | --- | --- | --- | --- | --- | --- | --- | --- | --- | --- | --- | --- | --- | --- |
|  | **Available for analyses** | | **Not available for analyses** | |  |  | **Available for analyses** | | **Not available for analyses** | |  |  | **Available for analyses** | | **Not available for analyses** | |  |
|  | **N = 196** | | **N = 68** | |  |  | **N = 125** | | **N = 46** | |  |  | **N = 215** | | **N = 99** | |  |
|  | **N** | **%** | **N** | **%** | *P value ^a^* |  | **N** | **%** | **N** | **%** | *P value ^a^* |  | **N** | **%** | **N** | **%** | *P value ^a^* |
| **Covariables** |  |  |  |  |  |  |  |  |  |  |  |  |  |  |  |  |  |
| Age: mean ±SD | 59.53 | **±**5.30 | 60.09 | **±**5.43 | *0.43* |  | 77.18 | **±**5.17 | 77.46 | **±**4.86 | *0.68* |  | 78.07 | **±**5.41 | 78.49 | **±**5.58 | *0.56* |
| Gender |  |  |  |  | *0.71* |  |  |  |  |  | *0.63* |  |  |  |  |  | *0.94* |
| Male | 28 | 14% | 11 | 16% |  |  | 23 | 18% | 7 | 15% |  |  | 77 | 36% | 35 | 35% |  |
| Female | 168 | 86% | 57 | 84% |  |  | 102 | 82% | 39 | 85% |  |  | 138 | 64% | 64 | 65% |  |
| Living conditions |  |  |  |  | *0.98* |  |  |  |  |  | *0.91* |  |  |  |  |  | *0.69* |
| Alone | 28 | 14% | 10 | 15% |  |  | 39 | 31% | 12 | 26% |  |  | 70 | 33% | 26 | 26% |  |
| With partner | 160 | 82% | 55 | 81% |  |  | 72 | 58% | 28 | 61% |  |  | 134 | 62% | 69 | 70% |  |
| With friends/family | 8 | 4% | 3 | 4% |  |  | 6 | 5% | 3 | 7% |  |  | 7 | 3% | 3 | 3% |  |
| Institutionalized | 0 | / | 0 | / |  |  | 8 | 6% | 3 | 7% |  |  | 4 | 2% | 1 | 1% |  |
| Cancer site |  |  |  |  | *0.46* |  |  |  |  |  | *0.53* |  |  |  |  |  |  |
| Breast | 150 | 77% | 49 | 72% |  |  | 93 | 74% | 32 | 70% |  |  |  |  |  |  |  |
| Colorectal | 46 | 23% | 19 | 28% |  |  | 32 | 26% | 14 | 30% |  |  |  |  |  |  |  |
| Cancer treatment |  |  |  |  |  |  |  |  |  |  |  |  |  |  |  |  |  |
| Surgery | 189 | 96% | 66 | 97% | *0.39* |  | 119 | 95% | 41 | 89% | *0.21* |  |  |  |  |  |  |
| Chemotherapy | 107 | 55% | 32 | 47% | *0.33* |  | 34 | 27% | 19 | 41% | *0.07* |  |  |  |  |  |  |
| Radiotherapy | 137 | 70% | 41 | 60% | *0.19* |  | 68 | 54% | 27 | 59% | *0.55* |  |  |  |  |  |  |
| Hormonal therapy | 108 | 55% | 41 | 60% | *0.39* |  | 53 | 42% | 17 | 37% | *0.56* |  |  |  |  |  |  |
| Targeted therapy | 18 | 9% | 3 | 4% | *0.22* |  | 4 | 3% | 2 | 4% | *0.71* |  |  |  |  |  |  |
| Unknown | 0 | / | 1 | 1% | */* |  | 1 | 1% | 1 | 2% | */* |  |  |  |  |  |  |
| Cancer Stage |  |  |  |  | *0.91* |  |  |  |  |  | ***0.01*** |  |  |  |  |  |  |
| I | 69 | 35% | 22 | 32% |  |  | 21 | 17% | 10 | 22% |  |  |  |  |  |  |  |
| II | 75 | 38% | 28 | 41% |  |  | 71 | 57% | 17 | 37% |  |  |  |  |  |  |  |
| III | 44 | 22% | 16 | 24% |  |  | 14 | 11% | 13 | 28% |  |  |  |  |  |  |  |
| Unknown | 8 | 4% | 2 | 3% |  |  | 19 | 15% | 6 | 13% |  |  |  |  |  |  |  |
| **Dependent variables** |  |  |  |  |  |  |  |  |  |  |  |  |  |  |  |  |  |
| Depression |  |  |  |  |  |  |  |  |  |  |  |  |  |  |  |  |  |
| Severity: mean ±SD | 1.86 | **±**2.28 | 1.89 | **±**1.99 | *0.56* |  | 2.15 | **±**2.30 | 1.68 | **±**1.98 | *0.17* |  | 1.78 | **±**2.03 | 2.45 | **±**2.53 | ***0.01*** |
| Frequency: ≥5 | 18 | 11% | 5 | 9% | *0.66* |  | 9 | 17% | 2 | 5% | *0.44* |  | 16 | 22% | 11 | 12% | *0.29* |
| Cognitive functioning |  |  |  |  |  |  |  |  |  |  |  |  |  |  |  |  |  |
| Severity: mean ±SD | 84.36 | **±**22.36 | 88.13 | **±**19.99 | *0.17* |  | 88.01 | **±**17.59 | 94.27 | **±**10.03 | *0.11* |  | 85.12 | **±**16.41 | 84.52 | **±**19.77 | *0.70* |
| Frequency: <67 | 54 | 28% | 14 | 21% | *0.30* |  | 16 | 18% | 2 | 6% | *0.11* |  | 47 | 22% | 22 | 22% | *0.92* |
| Fatigue |  |  |  |  |  |  |  |  |  |  |  |  |  |  |  |  |  |
| Severity: mean ±SD | 3.99 | **±**2.82 | 4.14 | **±**2.89 | *0.71* |  | 3.67 | **±**2.77 | 2.89 | **±**2.31 | *0.19* |  | 4.14 | **±**2.30 | 3.96 | **±**2.57 | *0.63* |
| Frequency: ≥3 | 98 | 52% | 41 | 62% | *0.17* |  | 42 | 53% | 13 | 46% | *0.54* |  | 120 | 57% | 57 | 58% | *0.83* |

*Note*: SD, standard deviation;

^a^ Differences between persons available for analyses versus persons lost to follow-up or those who skipped data collection at T1.
