# Supplementary material for: A cohort study on the evolution of psychosocial problems in older patients with breast or colorectal cancer: comparison with younger cancer patients and older primary care patients without cancer
Source: BMC Geriatr. 2015 Jul 9;15:79. doi: 10.1186/s12877-015-0071-7 (PMC4496825; doi:10.1186/s12877-015-0071-7)
Supplement: Additional file 2: Table S2. — Sensitivity analyses: best-case and worst-case scenario. [file 12877_2015_71_MOESM2_ESM.docx]

**Additional file 2: Table S2.** Sensitivity analyses: best-case and worst-case scenario

|  | **Depression**  **N = 453** | | | |  | **Cognitive functioning**  **N= 468** | | | |  | **Fatigue**  **N = 466** | | | |
| --- | --- | --- | --- | --- | --- | --- | --- | --- | --- | --- | --- | --- | --- | --- |
|  | **Best case** | | **Worst case** | |  | **Best case** | | **Worst case** | |  | **Best case** | | **Worst case** | |
|  | **OR** | **(95% CI)** | **OR** | **(95% CI)** |  | **OR** | **(95% CI)** | **OR** | **(95% CI)** |  | **OR** | **(95% CI)** | **OR** | **(95% CI)** |
|  |  |  |  |  |  |  |  |  |  |  |  |  |  |  |
| Baseline value of the problem | **9.69** | **(4.41 - 21.32)** | **7.51** | **(3.85 - 14.64)** |  | **4.29** | **(2.62 - 7.00)** | **3.25** | **(2.06 - 5.13)** |  | **3.05** | **(1.98 - 4.70)** | **2.98** | **(1.93 - 4.59)** |
|  |  |  |  |  |  |  |  |  |  |  |  |  |  |  |
| **Ageing-related variables** |  |  |  |  |  |  |  |  |  |  |  |  |  |  |
| Age: mean | 0.98 | (0.95 - 1.02) | 0.97 | (0.94 - 1.01) |  | 1.01 | (0.98 - 1.04) | 0.99 | (0.97 - 1.02) |  | 1.01 | (0.98 - 1.04) | 1.00 | (0.97 - 1.03) |
| Number of drugs: mean | 1.06 | (0.96 - 1.17) | 1.11 | (1.00 - 1.22) |  | 1.01 | (0.93 - 1.10) | 1.01 | (0.93 - 1.09) |  | **1.15** | **(1.06 - 1.25)** | **1.16** | **(1.06 - 1.26)** |
| Comorbidity: mean CCI score | 1.10 | (0.83 - 1.44) | 1.06 | (0.81 - 1.39) |  | 0.94 | (0.76 - 1.18) | 0.96 | (0.77 - 1.19) |  | 1.16 | (0.92 - 1.47) | 1.19 | (0.94 - 1.51) |
| Change in functional status |  |  |  |  |  |  |  |  |  |  |  |  |  |  |
| Not impaired | 1 | Reference | 1 | Reference |  | 1 | Reference | 1 | Reference |  | 1 | Reference | 1 | Reference |
| Was impaired | 1.89 | (0.54 - 6.58) | 1.94 | (0.56 - 6.76) |  | 1.62 | (0.73 - 3.60) | 1.54 | (0.70 - 3.37) |  | 1.79 | (0.90 - 3.58) | 1.52 | (0.76 - 3.03) |
| Became impaired | **3.93** | **(1.26 - 12.26)** | **4.43** | **(1.43 - 13.72)** |  | 1.93 | (0.89 - 4.20) | 1.73 | (0.80 - 3.74) |  | **2.30** | **(1.17 - 4.54)** | **2.11** | **(1.07 - 4.18)** |
| Persistently impaired | **7.08** | **(2.61 - 19.21)** | **8.06** | **(3.00 - 21.64)** |  | **2.40** | **(1.25 - 4.60)** | **2.19** | **(1.15 - 4.16)** |  | 1.46 | (0.84 - 2.54) | 1.41 | (0.81 - 2.47) |
|  |  |  |  |  |  |  |  |  |  |  |  |  |  |  |
| **Sociodemographic variables** |  |  |  |  |  |  |  |  |  |  |  |  |  |  |
| Gender (women) | 0.62 | (0.30 - 1.30) | 0.78 | (0.38 - 1.62) |  | 0.96 | (0.53 - 1.72) | 0.85 | (0.48 - 1.51) |  | **1.94** | **(1.12 - 3.36)** | 1.72 | (0.99 - 2.97) |
| Living conditions |  |  |  |  |  |  |  |  |  |  |  |  |  |  |
| With partner |  |  | 1 |  |  |  |  |  |  |  | 1 |  |  |  |
| With friends/family | 1.61 | (0.42 - 6.21) | 2.83 | (0.86 - 9.32) |  | 0.93 | (0.30 - 2.89) | 1.01 | (0.32 - 3.12) |  | 1.11 | (0.38 - 3.23) | 1.14 | (0.39 - 3.30) |
| Institutionalized | 1.66 | (0.26 - 10.59) | 2.20 | (0.41 – 11.73) |  | 0.20 | (0.02 - 1.86) | 0.19 | (0.02 - 1.75) |  | 0.66 | (0.13 - 3.31) | 0.53 | (0.11 - 2.60) |
| Alone | **2.24** | **(1.15 - 4.37)** | **2.19** | **(1.14 - 4.24)** |  | 1.01 | (0.59 - 1.75) | 1.02 | (0.60 - 1.74) |  | 0.88 | (0.53 - 1.47) | 0.94 | (0.56 - 1.57) |
| Age at leaving school |  |  |  |  |  |  |  |  |  |  |  |  |  |  |
| <15 years |  |  | 1 |  |  |  |  |  |  |  |  |  |  |  |
| 15 – 18 years | 0.77 | (0.37 - 1.63) | 0.94 | (0.45 – 1.96) |  | 1.14 | (0.62 - 2.09) | 1.20 | (0.66 - 2.19) |  | 1.52 | (0.88 - 2.65) | 1.54 | (0.88 - 2.69) |
| >18 years | 0.91 | (0.39 - 2.09) | 1.31 | (0.58 – 2.96) |  | 1.25 | (0.64 - 2.43) | 1.31 | (0.68 - 2.52) |  | 1.22 | (0.67 - 2.22) | 1.29 | (0.71 - 2.36) |
|  |  |  |  |  |  |  |  |  |  |  |  |  |  |  |
| **Cancer-related variables** |  |  |  |  |  |  |  |  |  |  |  |  |  |  |
| Cancer stage |  |  |  |  |  |  |  |  |  |  |  |  |  |  |
| No cancer |  |  | 1 |  |  |  |  |  |  |  |  |  |  |  |
| Surgery only | 0.76 | (0.20 - 2.95) | 1.39 | (0.46 - 4.23) |  | 0.91 | (0.33 - 2.50) | 0.70 | (0.26 - 1.90) |  | 0.68 | (0.29 - 1.56) | 0.48 | (0.21 - 1.12) |
| Surgery & RT/HT therapy or both | 2.31 | (0.96 - 5.53) | 1.65 | (0.71 – 3.85) |  | 1.55 | (0.79 - 3.06) | 1.10 | (0.56 - 2.17) |  | 1.21 | (0.65 - 2.27) | 0.85 | (0.45 - 1.60) |
| Surgery & CT with or without any combination^a^ | **3.62** | **(1.44 - 9.13)** | **2.53** | **(1.02 - 6.30)** |  | **2.10** | **(1.01 - 4.33)** | 1.47 | (0.72 - 3.01) |  | **2.00** | **(1.01 - 3.97)** | 1.38 | (0.70 - 2.75) |
| Other^b^ | / |  | / |  |  | 0.92 | (0.16 - 5.18) | 1.15 | (0.25 - 5.38) |  | 0.96 | (0.23 - 4.04) | 0.96 | (0.24 - 3.86) |
| **Percentage correctly classified** | 84% |  | 83% |  |  | 76% |  | 73% |  |  | 69% |  | 69% |  |
| **Goodness-of-fit test** | 0.76 |  | 0.96 |  |  | 0.18 |  | 0.19 |  |  | 0.29 |  | 0.34 |  |

*Note*: OR, Odds Ratio; 95% CI, 95% Confidence Interval; CCI, Charlson Comorbidity Index; RT, radiotherapy; HT, hormonal therapy; CT, chemotherapy; TT, targeted therapy

^a^ Surgery and CT with or without any combination RT, HT, TT

^b^ Other cancer treatments consisted of people who received no surgery but instead any of the following combinations: chemotherapy only (N = 2), chemo- and radiotherapy (N = 6), chemo- and targeted therapy (N=1), hormonal therapy only (N = 2)
