# Supplementary material for: A cohort study on the evolution of psychosocial problems in older patients with breast or colorectal cancer: comparison with younger cancer patients and older primary care patients without cancer
Source: BMC Geriatr. 2015 Jul 9;15:79. doi: 10.1186/s12877-015-0071-7 (PMC4496825; doi:10.1186/s12877-015-0071-7)
Supplement: Additional file 3: Table S3. — Frequency and severity of psychosocial problems at baseline and one-year follow-up for men and women separately. [file 12877_2015_71_MOESM3_ESM.docx]

**Additional file 3: Table S3.** Frequency and severity of psychosocial problems at baseline and one-year follow-up for men and women separately

|  | **WOMEN** | | | | | | | | | | **MEN** | | | | | | | | | | |
| --- | --- | --- | --- | --- | --- | --- | --- | --- | --- | --- | --- | --- | --- | --- | --- | --- | --- | --- | --- | --- | --- |
|  | **YCP** | | | *P-value^a^* | **OCP** | | *P-value ^b^* | **ONC** | | | **YCP** | | | *P-value^a^* | **OCP** | | | *P-value ^b^* | **ONC** | | |
| **DEPRESSION:** | **N=137** | | |  | **N=83** | |  | **N=127** | | | **N=26** | | |  | **N=18** | | |  | **N=65** | | |
| **Severity: mean (±SD)** |  |  | |  |  |  |  |  |  | |  |  | |  |  |  | |  |  |  | |
| Baseline | 1.93 | 2.40 | | *0.13* | 2.16 | 2.31 | *0.23* | 1.89 | 2.15 | | 1.46 | 1.48 | | *0.50* | 2.11 | 2.30 | | *0.43* | 1.55 | 1.76 | |
| After one year | 2.24 | 2.59 | | *0.18* | 2.47 | 2.30 | *0.18* | 2.04 | 2.18 | | 3.92 | 3.72 | | *0.06* | 1.72 | 2.22 | | *0.94* | 1.52 | 1.88 | |
| *P value within group difference* | *0.34* | | |  | *0.16* | |  | *0.39* | | | *0.00* | | |  | *0.40* | | |  | *0.74* | | |
| **Frequency: N (%)** |  |  | |  |  |  |  |  |  | |  |  | |  |  |  | |  |  |  | |
| Baseline | 17 | 12% | | *0.22* | 6 | 7% | *0.57* | 12 | 9% | | 1 | 4% | | *0.15* | 3 | 17% | | *0.16* | 4 | 6% | |
| After one year | 22 | 16% | | *0.54* | 16 | 19% | *0.19* | 16 | 13% | | 9 | 35% | | *0.08* | 2 | 11% | | *0.16* | 2 | 3% | |
| *P value within group difference* | *0.25* | | |  | *0.00* | |  | *0.29* | | | *0.00* | | |  | *0.56* | | |  | *0.16* | | |
|  |  | | |  |  | |  |  | | |  | | |  |  | | |  |  | | |
| **COGNITIVE FUNCTIONING:** | **N=168** | | |  | **N=68** | |  | **N=137** | | | **N=27** | | |  | **N=21** | | |  | **N=77** | | |
| **Severity: mean (±SD)** |  | |  |  |  |  |  |  | |  |  | |  |  |  | |  |  |  | |  |
| Baseline | 84.13 | | 22.69 | *0.57* | 87.50 | 17.60 | *0.19* | 85.64 | | 15.94 | 85.80 | | 20.52 | *0.63* | 89.68 | | 17.85 | *0.06* | 84.20 | | 17.29 |
| After one year | 82.84 | | 20.75 | *0.19* | 79.90 | 20.27 | *0.19* | 84.43 | | 15.68 | 77.16 | | 24.09 | *0.04* | 91.27 | | 12.49 | *0.11* | 83.98 | | 19.39 |
| *P value within group difference* | *0.29* | | |  | *0.00* | |  | *0.35* | | | *0.07* | | |  | *0.86* | | |  | *0.98* | | |
| **Frequency: N (%)** |  | |  |  |  |  |  |  | |  |  | |  |  |  | |  |  |  | |  |
| Baseline | 45 | | 27% | *0.22* | 13 | 19% | *0.56* | 31 | | 23% | 9 | | 33% | *0.13* | 3 | | 14% | *0.51* | 16 | | 21% |
| After one year | 48 | | 29% | 0.90 | 20 | 29% | 0.19 | 29 | | 21% | 12 | | 44% | *0.03* | 3 | | 14% | *0.37* | 18 | | 23% |
| *P value within group difference* | *0.67* | | |  | *0.07* | |  | *0.86* | | | *0.26* | | |  | *1.00* | | |  | *0.62* | | |
|  |  | | |  |  | |  |  | | |  | | |  |  | | |  |  | | |
| **FATIGUE:** | **N=160** | | |  | **N=59** | |  | **N=135** | | | **N=27** | | |  | **N=20** | | |  | **N=76** | | |
| **Severity: mean (±SD)** |  | |  |  |  |  |  |  | |  |  | |  |  |  | |  |  |  | |  |
| Baseline | 4.04 | | 2.86 | *0.65* | 3.83 | 2.87 | *0.21* | 4.39 | | 2.30 | 3.67 | | 2.57 | *0.52* | 3.20 | | 2.48 | *0.31* | 3.70 | | 2.25 |
| After one year | 4.44 | | 2.64 | *0.86* | 4.39 | 2.46 | *0.46* | 4.64 | | 2.22 | 4.52 | | 2.79 | *0.10* | 3.25 | | 2.45 | *0.23* | 4.03 | | 2.42 |
| *P value within group difference* | *0.18* | | |  | *0.19* | |  | *0.26* | | | *0.28* | | |  | *0.84* | | |  | *0.28* | | |
| **Frequency: N (%)** |  | |  |  |  |  |  |  | |  |  | |  |  |  | |  |  |  | |  |
| Baseline | 86 | | 54% | *0.95* | 32 | 54% | *0.35* | 83 | | 61% | 12 | | 44% | *0.71* | 10 | | 50% | *0.92* | 37 | | 49% |
| After one year | 95 | | 59% | *0.99* | 35 | 59% | *0.44* | 88 | | 65% | 17 | | 63% | *0.06* | 7 | | 35% | *0.11* | 42 | | 55% |
| *P value within group difference* | *0.24* | | |  | *0.51* | |  | *0.41* | | | *0.13* | | |  | *0.26* | | |  | *0.34* | | |

*Note*: Depression, cognitive functioning, and fatigue are presented as the mean score – indicated as the severity – and the proportion – indicated as frequency. Depression was measured with the 15-item Geriatric Depression Scale, range 0 – 15, higher scores indicate more depression, cut-off ≥5 for frequency of depression. Cognitive functioning was measured with the cognitive functioning subscale of the EORTC QLQ-C30, range 0 – 100, lower scores indicate worse functioning, cut-off <67 for frequency of cognitive impairment. Fatigue was measured with a Visual Analogue Scale, range 0 – 10, higher scores indicate more fatigue, cut-off ≥4 for frequency of fatigue.

^a^ Differences between older cancer patients and younger cancer patients

^b^ Differences between older cancer patients and older persons without cancer

**Additional table 4.** Frequency and severity of psychosocial problems at baseline and one-year follow-up for breast and colorectal cancer patients separately

|  | **Breast cancer** | | **Colorectal cancer** | | ***P-value^a^*** |
| --- | --- | --- | --- | --- | --- |
| **DEPRESSION:** | **N = 196** | | **N=68** | |  |
| **Severity: mean (±SD)** |  |  |  |  |  |
| Baseline | 2.02 | 2.34 | 1.84 | 2.11 | *0.64* |
| After one year | 2.36 | 2.56 | 2.69 | 2.89 | *0.61* |
| *P value within group difference* | *0.11* | | *0.05* | |  |
| **Frequency: N (%)** |  |  |  |  |  |
| Baseline | 20 | 10% | 7 | 10% | *0.98* |
| After one year | 36 | 18% | 13 | 19% | *0.89* |
| *P value within group difference* | *0.00* | | *0.11* | |  |
|  |  | |  | |  |
| **COGNITIVE FUNCTIONING:** | **N=208** | | **N=76** | |  |
| **Severity: mean (±SD)** |  |  |  |  |  |
| Baseline | 85.18 | 21.19 | 86.40 | 20.68 | *0.58* |
| After one year | 81.33 | 21.36 | 84.65 | 18.41 | *0.26* |
| *P value within group difference* | *0.01* | | *0.33* | |  |
| **Frequency: N (%)** |  |  |  |  |  |
| Baseline | 51 | 25% | 19 | 25% | *0.93* |
| After one year | 62 | 30% | 21 | 28% | *0.72* |
| *P value within group difference* | *0.14* | | *0.65* | |  |
|  |  | |  | |  |
| **FATIGUE:** | **N=192** | | **N=74** | |  |
| **Severity: mean (±SD)** |  |  |  |  |  |
| Baseline | 3.98 | 2.87 | 3.66 | 2.63 | *0.43* |
| After one year | 4.38 | 2.58 | 4.27 | 2.72 | *0.84* |
| *P value within group difference* | *0.15* | | *0.12* | |  |
| **Frequency: N (%)** |  |  |  |  |  |
| Baseline | 104 | 54% | 36 | 49% | *0.42* |
| After one year | 115 | 60% | 39 | 53% | *0.29* |
| *P value within group difference* | *0.19* | | *0.58* | |  |

*Note*: Depression, cognitive functioning, and fatigue are presented as the mean score – indicated as the severity – and the proportion – indicated as frequency. Depression was measured with the 15-item Geriatric Depression Scale, range 0 – 15, higher scores indicate more depression, cut-off ≥5 for frequency of depression. Cognitive functioning was measured with the cognitive functioning subscale of the EORTC QLQ-C30, range 0 – 100, lower scores indicate worse functioning, cut-off <67 for frequency of cognitive impairment. Fatigue was measured with a Visual Analogue Scale, range 0 – 10, higher scores indicate more fatigue, cut-off ≥4 for frequency of fatigue.

^a^ Differences between patients with breast and colorectal cancer
