# Supplementary material for: A cohort study on the evolution of psychosocial problems in older patients with breast or colorectal cancer: comparison with younger cancer patients and older primary care patients without cancer
Source: BMC Geriatr. 2015 Jul 9;15:79. doi: 10.1186/s12877-015-0071-7 (PMC4496825; doi:10.1186/s12877-015-0071-7)
Supplement: Additional file 4: Table S4. — Frequency and severity of psychosocial problems at baseline and one-year follow-up for breast and colorectal cancer patients separately. [file 12877_2015_71_MOESM4_ESM.docx]

**Additional file 4: Table S4.** Frequency and severity of psychosocial problems at baseline and one-year follow-up for breast and colorectal cancer patients separately

|  | **Breast cancer** | | **Colorectal cancer** | | ***P-value^a^*** |
| --- | --- | --- | --- | --- | --- |
| **DEPRESSION:** | **N = 196** | | **N=68** | |  |
| **Severity: mean (±SD)** |  |  |  |  |  |
| Baseline | 2.02 | 2.34 | 1.84 | 2.11 | *0.64* |
| After one year | 2.36 | 2.56 | 2.69 | 2.89 | *0.61* |
| *P value within group difference* | *0.11* | | *0.05* | |  |
| **Frequency: N (%)** |  |  |  |  |  |
| Baseline | 20 | 10% | 7 | 10% | *0.98* |
| After one year | 36 | 18% | 13 | 19% | *0.89* |
| *P value within group difference* | *0.00* | | *0.11* | |  |
|  |  | |  | |  |
| **COGNITIVE FUNCTIONING:** | **N=208** | | **N=76** | |  |
| **Severity: mean (±SD)** |  |  |  |  |  |
| Baseline | 85.18 | 21.19 | 86.40 | 20.68 | *0.58* |
| After one year | 81.33 | 21.36 | 84.65 | 18.41 | *0.26* |
| *P value within group difference* | *0.01* | | *0.33* | |  |
| **Frequency: N (%)** |  |  |  |  |  |
| Baseline | 51 | 25% | 19 | 25% | *0.93* |
| After one year | 62 | 30% | 21 | 28% | *0.72* |
| *P value within group difference* | *0.14* | | *0.65* | |  |
|  |  | |  | |  |
| **FATIGUE:** | **N=192** | | **N=74** | |  |
| **Severity: mean (±SD)** |  |  |  |  |  |
| Baseline | 3.98 | 2.87 | 3.66 | 2.63 | *0.43* |
| After one year | 4.38 | 2.58 | 4.27 | 2.72 | *0.84* |
| *P value within group difference* | *0.15* | | *0.12* | |  |
| **Frequency: N (%)** |  |  |  |  |  |
| Baseline | 104 | 54% | 36 | 49% | *0.42* |
| After one year | 115 | 60% | 39 | 53% | *0.29* |
| *P value within group difference* | *0.19* | | *0.58* | |  |

*Note*: Depression, cognitive functioning, and fatigue are presented as the mean score – indicated as the severity – and the proportion – indicated as frequency. Depression was measured with the 15-item Geriatric Depression Scale, range 0 – 15, higher scores indicate more depression, cut-off ≥5 for frequency of depression. Cognitive functioning was measured with the cognitive functioning subscale of the EORTC QLQ-C30, range 0 – 100, lower scores indicate worse functioning, cut-off <67 for frequency of cognitive impairment. Fatigue was measured with a Visual Analogue Scale, range 0 – 10, higher scores indicate more fatigue, cut-off ≥4 for frequency of fatigue.

^a^ Differences between patients with breast and colorectal cancer
